# Educational Approaches for Successful Implementation of interRAI Assessment Systems

**DOI:** 10.1177/08404704251390312

**Published:** 2025-11-10

**Authors:** Brigette Meehan, Mary James, Bonnie A. Pearson, John P. Hirdes

**Affiliations:** 1 637385 University of Otago, Dunedin North, New Zealand; 2 1259 University of Michigan, Ann Arbor, Michigan, United States of America; 3 Health eTraining International, Elora, Ontario, Canada; 4 8430 University of Waterloo, Waterloo, Ontario, Canada

## Abstract

Data from routine interRAI assessments can inform healthcare decisions at individual, organizational, and system levels. A well-designed, sustainable education strategy for all target audiences is essential to support both implementation and ongoing use of interRAI systems. Instructional design strategies and considerations for developing an educational program are described, as well as specific delivery suggestions for three audiences: (1) interRAI assessors who complete accurate assessments or coordinate them within teams; (2) clinicians who utilize interRAI assessment outcomes without conducting the assessments themselves; and (3) end users such as administrators, officials, and researchers who use interRAI data for management, governance, service delivery, performance measurement, policy, and research.

## Introduction

Globally, healthcare systems face challenges associated with population ageing, expansion of disability, and growing mental health concerns affecting all age groups.^1-5^ The complexity, prevalence, and duration of these conditions necessitate reconfiguration of services, redistribution of resources, policy changes, and operational reform for health systems to remain sustainable. An informed response to these challenges requires an evidence-base built on common data standards.^6,7^ A foundation of scientifically sound point-of-care data can maximize the benefits associated with new developments in personalized medicine, big data analytics, and artificial intelligence.^8^

In several countries, interRAI assessment and screening systems have been adopted as regional or national health data standards in long-term care, mental health services, and services targeting persons with disabilities.^9-18^ These systems have been implemented based on their well-established reliability and validity.^19-23^ In addition, the point-of-care data collected through interRAI instruments used across care settings can be employed to serve multiple applications for multiple stakeholders, including clinicians, administrators, policy-makers, and researchers.^12,17,24-29^ At the individual level, interRAI assessment systems support care planning, outcome measurement, prioritization of needs, service allocation, and coordination across settings and services to support individual autonomy, functioning, and well-being.^10,13,30^

An effective, comprehensive, and ongoing educational strategy is foundational for successful implementation of interRAI assessment instruments. A fundamental question is whether data obtained from routine use can be employed to serve multiple applications in clinical practice, performance measurement, funding allocation, and policy development. Research has shown that data from routine use of interRAI systems meets all appropriate standards for reliability and validity.^10,31-33^ To achieve those standards, a carefully planned, comprehensive, and robust education strategy is essential for all audiences using interRAI-based evidence to inform their decision-making.^12,34-37^ Key target audiences should include not only interRAI assessors and other clinicians using the information to formulate a person’s care plan and track outcomes of care, but also health leaders who use the aggregated data for management, planning, policy development, and research. This article is a narrative review of the conceptual, strategic, and operational considerations for an educational strategy to support implementation and ongoing use of interRAI systems, from general design strategies to specific delivery suggestions for specific stakeholders. No human subjects were involved, and ethics clearance was not required for this review.

## Addressing the Priorities and Interests of Key Target Audiences for interRAI Education

There are three main audiences for interRAI education each with unique information needs, interests, and roles in implementation of these systems. A comprehensive educational strategy should address these differences while also fostering a shared vision of improved quality of care as the basis for interRAI implementation.

The most intensive education is aimed at clinicians responsible for completing accurate interRAI assessments, either by themselves or by coordinating a team-based approach. Their work forms the foundation for all uses of interRAI data across the health system. For this group, the educational focus includes the technical understanding of interRAI item intents, definitions, documenting and coding rules, and processes to ensure assessments are completed in a correct and timely manner. Assessors are trained to exercise professional judgment, using all sources of information to identify the clinical status, strengths, preferences, and needs of those assessed, including conversations with and observations of individuals and their caregivers, consultation with colleagues, and review of clinically relevant documentation. Problem solving skills, observation and interviewing techniques, attitudes, and communication strategies are broader components that are vital to the educational program.

Clinicians who *use* interRAI assessment results (but do not complete the assessments) are a second major target group. They must understand and trust how assessors conduct assessments and complete interRAI items. Moreover, the interpretation and clinical application of assessment results (e.g., care planning and outcome measurement) is essential for this group. For interRAI assessments to be more than an organizational reporting requirement, clinical partners must (a) be confident in using assessment information completed by others; (b) support assessors by sharing information pertinent to the assessment(s); (c) use assessment results in the clinical team’s deliberations; and (d) engage care recipients and their support network in a collaborative approach to decision-making informed by interRAI assessment results.

A third group needing interRAI education includes administrators, government officials, and researchers making secondary use of interRAI data for management, governance, service delivery, quality improvement, performance measurement, policy development, and scientific inquiry. These audiences need a broader based understanding of interRAI systems that ensures clarity in their interpretation of interRAI items, scales, and decision-support tools. These groups must also embrace a leadership role in compiling and communicating key messages and providing resources to assessors to establish a positive collaborative environment that engages all partners in interRAI assessment. For example, Hikaka et al. (2024)^11^ describe leadership by New Zealand researchers who established a collaborative national network to support knowledge translation of interRAI research and provide evidence to inform policy development and health system improvement. Similarly, the Seniors Quality Leap Initiative is a community of practice that supports use of interRAI-based indicators to improve quality within long-term care settings.^12^

**Figure 1.**
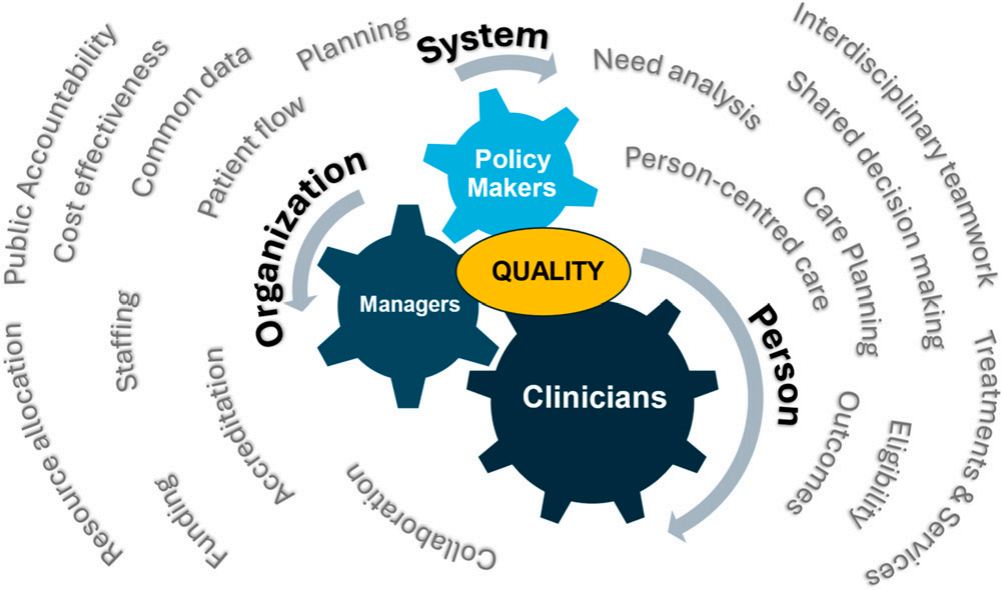
Intersecting Relationships Between Internal Motivations of Clinician Learners and External Motivations of Managers and Policy-Makers

Hidi and colleagues^38-40^ note that learners may have immediate internal interests that are not always the same as those of external parties in their work environment. In practical terms, this means that some motivations that managers and government officials have for implementing interRAI systems may not be of interest to clinicians completing the assessments (see Figure 1). Clinical assessors are often most interested by the clinical and person-level applications of interRAI systems (e.g., care planning). Managers usually focus on organization-level applications (e.g., accreditation and staffing), whereas governments may be most interested in system or population-level uses (e.g., equity, public accountability, and cost-effectiveness). However, the central unifying theme that can be a common motivation for all parties is quality of care. A shared vision of “making things better” can be achieved using the quality applications of interRAI systems as a foundation for education at all levels.

## Principles for Design of interRAI Education

A fully realized implementation of interRAI systems that enhances quality frameworks such as the Institute for Healthcare Improvement’s Quintuple Aim^41^ does not happen by chance. It requires a carefully considered strategy that embeds a continuous approach to learning as part of professional practice with the use of interRAI systems. An effective strategy considers instructional design, collaboration, competency evaluation, and establishment of an educational ecosystem to support continuous learning.

## Motivational Design

Instructional design involves development of goals and objectives of education; specification of instructional strategies; development of learning materials; delivery of in-person, online, or hybrid instruction; and evaluation of learning outcomes.^42^ These aspects of design focus on the cognitive realm of learning; however, the affective considerations of learning are equally important. What you are expected to learn (cognitive) and how you feel about the learning experience (affective) are two inter-related components of educational experiences.

Motivational design considers both the development of materials that are motivating and approaches to facilitating the intrinsic motivation of the learner. Keller described this approach as a general framework for learning,^43^ and it has been used successfully in health education.^43-47^

**Figure 2.**
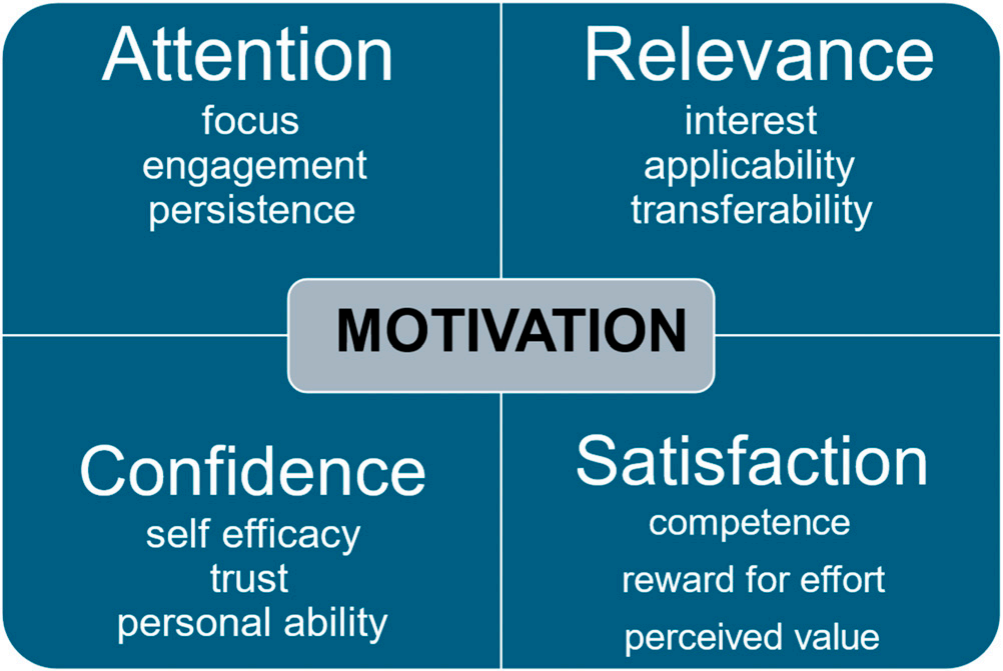
Schematic Summary of Key Dimensions of Motivation Design Based on the ARCS Model^43^

Figure 2 shows the four main dimension of motivational design.

**Attention** considers the extent to which the learner can focus on educational tasks, feels engaged in the learning process, understands the necessity of learning this content, and persists with task completion in the various stages of learning. Examples of how this can be achieved include protection of work time for learning and monitoring progress through learner materials.

**Relevance** encompasses the learner’s level of interest in the learning opportunity and materials, ensuring the relevance of the material to the learner’s work responsibilities, and transferring knowledge and skills learned to the workplace. This can be achieved with realistic case studies for both coding interRAI assessments and interpreting assessment results, relevance checking, effective use of media, use of infographics, and use of engaging applicable content.

**Confidence** pertains to the learner’s self-efficacy regarding their expectation that they will succeed in the learning process, trust in the veracity and utility of the educator and the learning materials, and the learner’s sense that they have the personal ability to understand and use the information being taught. For example, educators can facilitate a positive learning environment by ensuring instruction materials are clear, e-learning software functions appropriately and intuitively, and the pace of learning is appropriate for the learner’s ability and the complexity of the content. In addition, opinion leaders and mentors from different disciplines can be engaged to model their own personal growth and success in application of interRAI systems.

**Satisfaction** considers whether the person has achieved an expected level of knowledge, skill, and competence at a given stage of learning. It also includes the learner’s sense that the effort expended is rewarded by a concomitant level of personal and professional growth and that the person values having gone through the learning experience. Competency testing, inclusion of learning achievements in professional performance reviews, and positive reinforcement by peers and managers can all contribute to this dimension.

## Co-Design of Educational Content and Opportunities

Learning to complete and use information from interRAI systems requires co-design of all aspects of the educational strategy for clinicians, managers, policy-makers, and researchers. While experts need to develop accurate educational content about interRAI systems, co-design will help integrate projects into the organization.^48^ Co-design provides learners with opportunities to identify their interests, learning needs, and priorities for education. They can also provide educators with insights about relevant case examples, identify organizational facilitators and barriers to learning, and guide changes to internal processes to embed new clinical and management practices informed by interRAI-based evidence. In addition, co-design supports the development of organizational champions who are vital to internal implementation efforts.

## Competency-Based Learning Strategies

The educational program must establish objectives and competencies whereby the learner demonstrates the ability to apply the new competencies to the task or functions required for their role. Knowledge, skills, and attitudes are important components of a competency statement^49,50^ which defines the content to be learned, the intended demonstrable outcome, agreed activities to embed training, and delineation of how competency will be applied and measured. In this instance knowledge includes interRAI assessment, terminology, and accurate coding. Skills involve observation, conversation, and interview techniques, integrating varied information sources through the software, analyzing findings, interpreting outcomes, communicating effectively, care planning, and decision-making. Attitude comprises engagement, active collaboration in assessment, valuing shared decision-making, respecting sensitivity around personal information, and taking ownership of the clinical process.

## Establishing an Educational Ecosystem for interRAI Implementation

**Figure 3.**
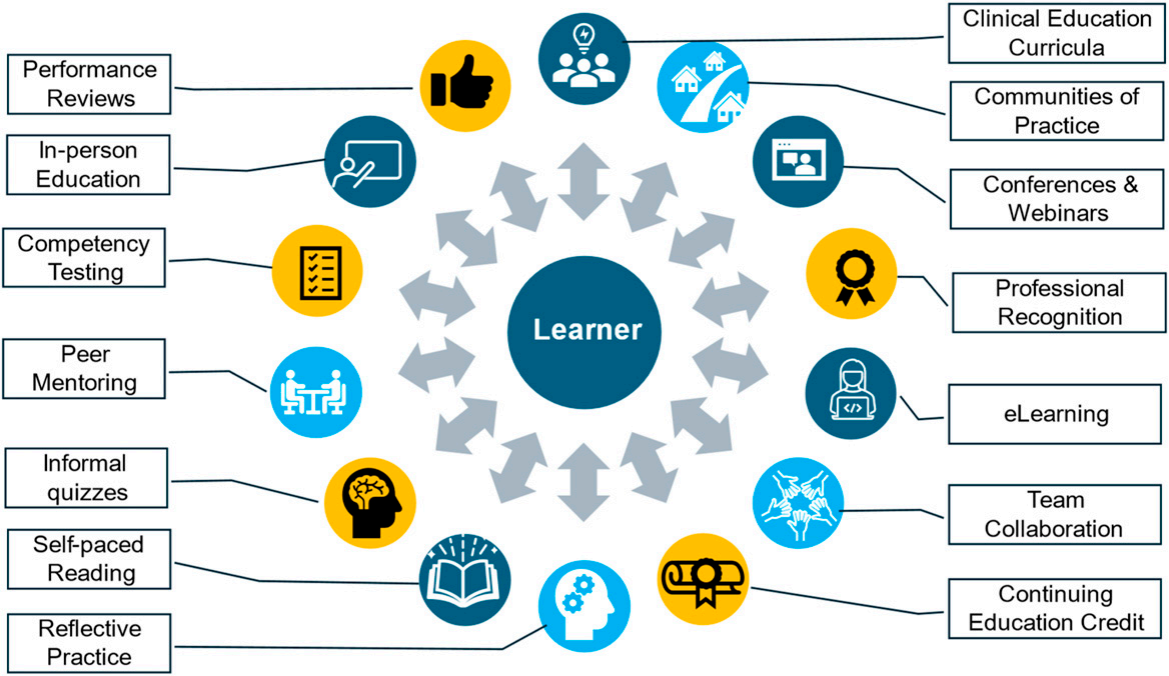
An Education Ecosystem to Support Effective Implementation of interRAI Systems

There is no single approach to training that can fulfil all educational needs of all learners in the health system to provide all stages of knowledge and skill ascertainment needed for successful implementation of interRAI systems. As Figure 3 shows, an effective strategy involves the strategic establishment of an educational ecosystem to support diverse educational needs in the implementation and ongoing use of interRAI systems. This ecosystem involves at least three main inter-related dimensions.

First, the ecosystem must include diverse mechanisms for knowledge ascertainment. This includes ensuring learners have direct knowledge of and access to interRAI assessment manuals and continue to read materials from professional peer reviewed sources regarding interRAI systems. In-person, e-learning, and hybrid solutions can be used to teach coding rules, scale interpretation, assessment guidelines, and use of interRAI decision-support tools. In addition, professional webcasts, conferences, and webinars provide opportunities to engage with interRAI experts, clinical leaders, and other interRAI users.

Second, there should be a variety of learning tools used to acquire knowledge, and to evaluate, monitor, and recognize achievements in all aspects of interRAI system use. This can include case review, informal self-guided testing in educational modules, competency testing, provision of continuing education credits, inclusion of interRAI education in performance evaluations, and professional recognition for leadership and innovation in use of interRAI systems.

Third, an effective education ecosystem includes support and cultural factors to engage learners and users to use interRAI systems. At the person level, clinicians and managers should be encouraged to engage in reflective practice on how daily professional use of interRAI systems relates to improving the quality of care with a person-centred approach. Teamwork to ensure all learners are supported and one-to-one mentoring are essential for problem solving, building confidence, and confirming the value of assessments for the individual care recipient and within the organization. Communities of practice can enhance the positive impact of interRAI systems at the program, unit, and organization level and peer organizations can collaborate to learn from each other’s experiences to reduce challenges and maximize chances of success.

## Education Delivery Models

A variety of models are available for delivering education. For clinical assessor training, the traditional method involves face-to-face instruction utilizing a “train-the-trainer” approach.^13,37^ In this model, external educators from recognized interRAI training organizations instruct in-house practice leaders, who then assume the role of educators within their own departments or organizations. Alternatively, an experienced external educator may be engaged for a defined period to provide training, during which time suitable in-house “champions” are identified to offer ongoing peer support.

Technological advancements have expanded educational options, enabling hybrid models that combine in-person education with online self-directed study, as well as fully online programs integrating independent learning with educator-guided sessions. New opportunities in AI-based education are emerging; however, these will continue to require educator oversight to ensure accuracy and appropriateness of artificial intelligence-based content. Learner engagement and sustainability remain key considerations when selecting appropriate delivery models, given that learners from all target audiences will change over time, and those who remain require continuous support to maintain or enhance their understanding of the interRAI assessment system.

Groups such as informal caregivers and advocacy organizations may benefit more from written materials or video presentations in accessible languages, designed to clarify interRAI methodology and its associated value. Additionally, infographics, dynamic data visualization, or self-paced learning modules can serve as effective tools for educating organizational leaders, business managers, policy-makers, and planners, supporting them in leveraging the assessment system to achieve their strategic and operational objectives.

## Gaps and Limitations

The paucity of empirical evidence regarding the relative effectiveness of different educational approaches is an important gap in the literature. Turcotte et al.^36^ demonstrated the use of statistical tools to evaluate the effectiveness of interRAI training on data quality; however, the lack of an international and multi-sectoral body of evidence regarding educational approaches is an important limitation to be addressed in future research.

## Conclusion

Education is key to the successful implementation of interRAI assessment systems. Implementation is complex due to the diversity in target audiences, each with specific interests in comprehending and utilizing quality data within their respective domains. However, with appropriate resources and leadership a dynamic educational ecosystem can evolve to support the full use of interRAI systems to improve health system performance. Tailored educational strategies must go beyond accurate completion of a form to include enhancing individual health outcomes, improving system performance, population planning, funding allocation, public accountability, equity, and expanding knowledge with application of research findings. Effective organizational leadership is essential to address both strategic and operational considerations regarding interRAI assessment education for assessors and partners in decision-making.
